# A reference-grade genome assembly data of sika deer in Hokkaido, Japan, *Cervus nippon yesoensis*

**DOI:** 10.1016/j.dib.2025.112423

**Published:** 2025-12-27

**Authors:** Yuki Matsumoto, Junco Nagata, Yukiko Matsuura, Hayato Iijima

**Affiliations:** a Research and Development Section, Anicom Specialty Medical Institute Inc., 2-6-3 5F Chojamachi, Yokohamashi-Nakaku, Kanagawa 231-0033, Japan; b Data Science Center, Azabu University, 1-17-71 Fuchinobe, Chuo-ku, Sagamihara-shi, Kanagawa 252-5201, Japan; c Forestry and Forest Products Research Institute, 1 Matsunosato, Tsukuba, Ibaraki 305-8687 Japan; d Hokkaido Research Center, Forestry and Forest Products Research Institute, 7 Hitsujigaoka, Toyohira, Sapporo, Hokkaido 062-8516, Japan

**Keywords:** Cervidae, De novo genome assembly, Long-read sequencing, Genomic diversity

## Abstract

Sika deer (*Cervus nippon*) is naturally distributed across East Asia and includes 14 subspecies, showing phenotypic and genetic diversity. In this study, we constructed a de novo genome assembly of wild sika deer using one of the largest subspecies, *C. n. yesoensis*. We used HiFi, high quality long-read based on Pacific Bioscience to assemble our novel genome assembly CerNipYes1.0. The genome size of CerNipYes1.0 is estimated to be 3.1Gb, which is 0.6Gb larger than the other genome assembly of sika deer previously reported. The number of scaffolds is 1,810 and N50 length achieved 77Mb. Compleasm, a genome completeness evaluation tool based on Benchmarking Universal Single-Copy Orthologs (BUSCO) indicated that 12,562 (99.75%) genes are completed as genes with comparing to database. Our results indicate that CerNipYes1.0 is valuable to study the molecular biology, phylogeny and evolution of the Cervidae and its genome.

Specifications Table

**Table:** 

Subject	Biology
Specific subject area	*Bioinformatics, Genomics*
Type of data	*Raw, Filtered, and Processed.*
Data collection	Genomic DNA was extracted from a muscle sample of sika deer using the NucleoBond® AXG Column (Takara Bio, Inc.). Genomic libraries were constructed with the SMRTbell® Express Template Prep Kit 2.0 (Pacific Biosciences, California, USA), and long-read HiFi sequencing was performed on the PacBio Sequel II platform according to the manufacturer's protocol. Sequencing data were generated using SMRT Link v8.0.0 (Pacific Biosciences), and genome assembly was conducted using Hifiasm v0.19.5, followed by scaffolding with RagTag v2.1.0.
Data source location	• Institution: Research and Development Section, Anicom Specialty Medical Institute Inc. • City/Town/Region: 2–6–3 5F Chojamachi, Yokohamashi-Nakaku, Kanagawa 231–0033 • Country: Japan • Latitude and longitude for collected samples/data: 44.27470° N, 142.97850° E.
Data accessibility	Raw data Repository name: DDBJ Data identification number: PRJDB35526; DRR702133 Direct URL to data: https://ddbj.nig.ac.jp/search/entry/bioproject/PRJDB35526; https://ddbj.nig.ac.jp/search/entry/sra-run/DRR702133.
Related research article	*none*

## 1 Value of the Data

• The reference-grade genome assembly of wild sika deer from Hokkaido, Japan (*Cervus nippon yesoensis*), constructed using PacBio HiFi sequencing, has been made publicly available.
• Completeness of genes indicates that CerNipYes1.0. is higher quality than another sika deer genome, MHL_v1.0, and that the assembly is the second-highest quality in comparison to others, while the highest one is mCerEla1.1
• Considering the diversity of the genome size and phenotypic traits among Cervidae species, the genome resources provided here can offer valuable information to support further studies for molecular biology, phylogeny and evolution of the Cervidae.

## 2 Background

Cervidae is the second largest family of the terrestrial artiodactyls, consisting of over 50 extant species [1] and representing the major part of terrestrial mammal biomass [2]. One of these species, the sika deer (*Cervus nippon*), is naturally distributed across East Asia and includes 14 subspecies [3]. One of the largest subspecies, *C. n. yesoensis* distributed in Hokkaido, Japan, has been well studied based on ecological [4] and management context [3], and genetic studies have suggested a recent population expansion in distribution after a severe bottleneck [5,6]. Whole genome sequencing has become popular to explore population dynamics, phylogeny, and/or molecular mechanism underlie phenotypic traits [7,8]. To date, the genomes of six Cervus individuals have been assembled (Table 1, [7,[9], [10], [11], [12], [13]]). The previously reported sika deer genome [7] was assembled using an individual from a deer farm in China, and its subspecies was unknown. Therefore, neither wild sika deer nor the sika deer native to Japan have been whole genome sequenced so far. In this study, we conducted genome assembly using long-read sequencing technology to investigate genome structure of wild sika deer from Hokkaido, Japan, whose origin and subspecies are known.

**Table 1:** Comparative metadata of genome assemblies utilized in the current study.

Species	Common name	Assembly	Isolate or specimen ID	Sex	Sequencing Methods	Core assembly method	Study
**Cervus nippon yesoensis**	**Sika deer**	**CerNipYes1.0**	**JP_HOKNOK_M01**	**male**	**PacBio HiFi**	**Hifiasm**	**This study**
Cervus nippon	Sika deer	MHL_v1.0	Not available	female	PacBio; Illumina; Hi-C	wtdbg	Xing et al. GPB 2023
Cervus albirostris	White-lipped deer	WLD	WLD-bcl	not collected	Illumina HiSeq	platanus	London et al. J Hered 2022
Cervus hanglu yarkandensis	Tarim red deer	CEY_v1	CEY-2017	male	10X Genomics; Illumina NextSeq	Supernova	Ba et al. Sci data 2020
Cervus canadensis	Wapiti	ASM1932006v1	Bull #8	male	PacBio RSII; Illumina HiSeq	MaSuRCA	Masonbrink et al. PLOS ONE 2021
Cervus elaphus	Red deer	mCerEla1.1	SAN0000996	female	PacBio; 10X Genomics; Hi-C	Hifiasm	Pemberton et al. Wellcome Open Res. 2020
Cervus elaphus hippelaphus	Red deer	CerEla1.0	Hungarian	male	Illumina HiSeq	AllPaths	Vozdova et al. Animals 2021

Bold indicates the assembly developed in the current study.

## 3 Data Description

A de novo genome assembly of *C. n. yesoensis* was constructed using long-read HiFi sequencing based on Pacific Biosciences (PacBio) platform (Sequel II) (DDBJ accession: PRJDB35526 and DRR702133). Hifiasm using the HiFi reads yielded a de novo genome assembly, and after scaffolding we constructed CerNipYes1.0 (NCBI accession: JBTNMK000000000). We obtained 3,946,389 reads and sequenced 58,855,465,335 bp in total. The average read length was 14,913.8 bp. The estimated coverage depth was approximately 18.7x. We compared the genome assembly of six Cervidae species (Table 1). The genome size of CerNipYes1.0 is estimated to be 3.1Gb, which is 0.6Gb larger than the other genome assembly of sika deer previously reported. The number of scaffolds is 1810 and N50 length achieved 77Mb (Table 2). The estimated genome size varies among seven assemblies, suggesting existing chromosome variation in Cervidae. In order to confirm the quality of CerNipYes1.0 as the genome assembly, we used compleasm [14], which is an efficient tool for assessing the completeness of genome assemblies using the miniprot protein-to-genome aligner and conserved orthologous genes from BUSCO [15]. One of the statistics of compleasm to calculate completeness of genes (C), indicates that CerNipYes1.0. is higher quality than another sika deer genome, MHL_v1.0, and that the assembly is the second-highest quality in comparison to others, while the highest one is mCerEla1.1 (Table 3). The RepeatMasker analysis of the assembled genome, CerNipYes1.0, revealed that the total content of repetitive elements was 21.63 %, which is comparable to that of other related assemblies (ranging from 14.46 % to 25.39 %) (Table 4).

**Table 2:** Standard sequence and contiguity metrics in each genome assembly.

Common name	Assembly	Total length (bp)	Number	Longest	Shortest	N count	Gaps	N50	N50n
**Sika deer**	**CerNipYes1.0**	**3,147,430,571**	**1,810**	**159,839,349**	**12,800**	**57,300**	**573**	**77,397,291**	**15**
Sika deer	MHL_v1.0	2,500,646,934	588	143,481,735	2,912	145,300	1,453	78,786,809	12
White-lipped deer	WLD	2,692,225,130	171,874	23,094,547	201	50,310,236	268,196	3,769,372	218
Tarim red deer	CEY_v1	2,594,114,534	17,927	134,243,713	1,000	33,508,101	21,781	77,688,133	13
Wapiti	ASM1932006v1	2,526,613,007	185	146,388,637	1,015	1,906,222	6,643	77,654,944	13
Red deer	mCerEla1.1	2,886,603,524	144	179,953,079	16,426	10,400	40	83,473,711	13
Red deer	CerEla1.0	3,438,623,608	11,479	181,543,317	222	1477,834,197	435,163	107,358,006	13

Bold indicates the assembly developed in the current study.

**Table 3:** Completeness of genome assemblies using compleasm (12,594 genes).

Common name	Assembly	C (S + D)	S	D	F	I	M
**Sika deer**	**CerNipYes1.0**	**12,562**	**12,119**	**443**	**20**	**0**	**12**
Sika deer	MHL_v1.0	12,500	12,315	185	25	0	69
White-lipped deer	WLD	12,006	11,832	174	406	0	182
Tarim red deer	CEY_v1	12,501	12,169	332	74	0	19
Wapiti	ASM1932006v1	12,415	12,187	228	55	0	124
Red deer	mCerEla1.1	12,568	12,344	224	21	0	5
Red deer	CerEla1.0	10,654	10,605	49	996	1	943

Bold indicates the assembly developed in the current study.

**Table 4:** Repeat plofile of genome assemblies using RepeatMasker.

Common name	Assembly	Element	number of elements	length occupied (bp)	percentage of sequence
**Sika deer**	**CerNipYes1.0**	**SINEs**	**1,228,518**	**141,895,774**	**4.51 %**
		**Penelope**	**864**	**72,158**	**0.00 %**
		**LINEs**	**786,516**	**375,556,939**	**11.93 %**
		**LTR elements**	**260,930**	**89,518,696**	**2.84 %**
		**DNA elements**	**450,553**	**73,357,270**	**2.33 %**
		**Unclassified**	**3,034**	**463,574**	**0.01 %**
		**Total interspersed repeats**		**680,864,411**	**21.63 %**
Sika deer	MHL_v1.0	SINEs	1,175,543	135,749,356	5.43 %
		Penelope	845	71,259	0.00 %
		LINEs	747,953	354,732,713	14.19 %
		LTR elements	245,252	85,000,380	3.40 %
		DNA elements	295,565	57,817,447	2.31 %
		Unclassified	2,876	450,955	0.02 %
		Total interspersed repeats		633,822,110	25.35 %
White-lipped deer	WLD	SINEs	1,218,414	140,153,643	5.21 %
		Penelope	818	69,706	0.00 %
		LINEs	795,785	358,868,372	13.33 %
		LTR elements	256,781	87,843,332	3.26 %
		DNA elements	305,490	59,408,016	2.21 %
		Unclassified	2,965	460,643	0.02 %
		Total interspersed repeats		646,803,712	24.02 %
Tarim red deer	CEY_v1	SINEs	1,198,926	138,350,708	5.33 %
		Penelope	843	71,773	0.00 %
		LINEs	771,045	365,514,802	14.09 %
		LTR elements	250,987	87,150,863	3.36 %
		DNA elements	301,529	59,076,765	2.28 %
		Unclassified	2,935	457,527	0.02 %
		Total interspersed repeats		650,622,438	25.08 %
Wapiti	ASM1932006v1	SINEs	1,182,147	136,508,425	5.40 %
		Penelope	831	69,820	0.00 %
		LINEs	757,197	360,608,658	14.27 %
		LTR elements	246,981	85,656,270	3.39 %
		DNA elements	296,632	58,111,078	2.30 %
		Unclassified	2,886	451,411	0.02 %
		Total interspersed repeats		641,405,662	25.39 %
Red deer	mCerEla1.1	SINEs	1,199,359	138,496,239	4.80 %
		Penelope	843	70,596	0.00 %
		LINEs	769,666	372,508,590	12.90 %
		LTR elements	253,405	87,350,595	3.03 %
		DNA elements	414,312	68,936,441	2.39 %
		Unclassified	2,939	458,106	0.02 %
		Total interspersed repeats		667,820,567	23.14 %
Red deer	CerEla1.0	SINEs	993,366	114,508,777	3.33 %
		Penelope	659	56,146	0.00 %
		LINEs	643,677	259,633,294	7.55 %
		LTR elements	219,382	72,834,881	2.12 %
		DNA elements	259,584	49,902,378	1.45 %
		Unclassified	2,598	404,951	0.01 %
		Total interspersed repeats		497,340,427	14.46 %

Bold indicates the assembly developed in the current study.

## 4 Experimental Design, Materials and Methods

### 4.1 Sample collection

We obtained a male sika deer (JP_HOKNOK_M01) captured in Nishiokoppe Village, where there is a special hunting area authorized by the Hokkaido governor, Hokkaido, Japan. The adult male deer was culled legally in 2022 to prevent agricultural damage. The collection site was located at 44.27470° N, 142.97850° E.

### 4.2 DNA extraction and sequencing

A muscle sample from the sika deer was used to extract DNA using NucleoBond® AXG Column (Takara Bio, Inc.). The high molecular genomic DNA was physically fragmented to approximately 20 kb using Megaruptor 3. After blunting both ends of the fragmented DNA, 3′-dA protruding ends were treated, and SMRTbell adapters were ligated to create a sequencing library that serves as a sequencing template using SMRTbell Express Template Prep Kit 2.0 and Barcoded Overhang Adapter Kit (Pacific Biosciences Inc.). The entire volume of the resulting sequencing library was mixed, the size distribution was confirmed by electrophoresis, and size selection was performed using SageELF (Sage Science Inc.).

Sequencing primers complementary to both ends of the SMRTbell™ adapters and DNA polymerase were annealed to the sequencing library to form sequencing templates. These templates were loaded onto a SMRT® Cell, where they were immobilized within zero-mode waveguides (ZMWs), and sequencing reactions were performed using PacBio Sequel II System.

### 4.3 Genome assembly

A quality control was performed using fastp v.0.22.0 with quality score exceeding 30 [16]. The reads were filtered and merged into one fastq file. Then we used Hifiasm v 0.19.5 [17] with default settings for de novo assembly using the fastq file. After generating the de novo assembly, we used RagTag v2.1.0 [18] to scaffold the mCerEla1.1 genome, which is the highest genome assembly in 6 deer species compared in this study. The six deposited genome assemblies were downloaded from NCBI Genbank (https://www.ncbi.nlm.nih.gov/datasets/genome). We compared genome assembly statistics, such as total length (genome size) and number of scaffolds/contigs, using assembly-stats v 1.0.1[19]. An assessing tool to test the completeness of genome assembly, compleasm v0.2.7, a genome completeness evaluation tool based on Benchmarking Universal Single-Copy Orthologs (BUSCO), is used for checking the quality based on orthologous genes conserved across a wide range of taxa. The software runs with artiodactyla_obd12 datasets with default settings (12,594 genes in total). Repetitive element annotations were assessed using RepeatMasker version 4.2.2 with rmblastn version 2.14.1+ and FamDB (CONS-Dfam_3.9; Cervus elaphus).

## Limitations

The total number of scaffolds is 1,810, although the chromosome number of the species is 2n=68 [20]. This indicated our data has not completed construction at the chromosome-scale. No chromosome and gene annotation were performed. Future study will require complete sequencing and annotating the mitochondrial genome and Y chromosomes, in addition to the gene annotation.

## Ethics Statement

The work meets the ethical requirements for publication in Data in Brief.

## CRediT authorship contribution statement

**Yuki Matsumoto:** Conceptualization, Project administration, Methodology, Writing – original draft, Writing – review & editing, Data curation. **Junco Nagata:** Supervision, Methodology, Writing – review & editing. **Yukiko Matsuura:** Methodology, Writing – review & editing. **Hayato Iijima:** Supervision, Methodology, Writing – review & editing, Project administration, Funding acquisition.

## Data Availability

National Center for Biotechnology InformationCervus nippon yesoensis isolate:Nishiokoppe Village Genome sequencing (Reference data).
